# Analysis of medication consultation networks and reporting medication errors: a mixed methods study

**DOI:** 10.1186/s12913-018-3049-2

**Published:** 2018-03-27

**Authors:** Pattarida Janmano, Uraiwan Chaichanawirote, Chuenjid Kongkaew

**Affiliations:** 1 Department of Pharmacy, Phrasamutchedisawatyanon Hospital, Samutprakan, 10290 Thailand; 2 Faculty of Nursing, Naresuan University, Phitsanulok, 65000 Thailand; 3 Research Centre for Safety and Quality in Health, Faculty of Pharmaceutical Sciences, Naresuan University, Phitsanulok, 65000 Thailand; 4 Research Department of Practice and Policy, School of Pharmacy, University College London, London, UK

**Keywords:** Medication error, Social network analysis, Medication safety

## Abstract

**Background:**

To examine characteristics of verbal consultation about medication within social networks of hospital inpatient medication system, and their associations with medication error reporting.

**Method:**

The setting was a 90-bed provincial district hospital with 4 wards, 7 physicians, 5 pharmacists, 44 nurses, 5 pharmacist assistants, and 4 unskilled ancillary workers. A mixed method comprising (i) a prospective observational study for investigating incidences and the nature of reporting medication errors, and (ii) a social network analysis for patterns of interaction.

**Results:**

Out of 5296 prescriptions, 132 medication errors were reported during the one month study period: an incidence rate of 2.5%. Every incident of medication errors was formally documented through pharmacists. The most frequent medication errors were in pre-transcribing (*n* = 54; 40.9%). The pharmacists were central in the whole network of consultation on medication with the mean in-degree centrality of 35 (SD 14.9) and mean out-degree centrality of 15.4 (SD 11.1). Two bridging participants were identified who were influential communicators connecting the network (betweenness centrality). Medication error reporting were influenced by (i) participants whose advice is sought and viewed as trustworthy (in-degree centrality; *p* < 0.001), (ii) sex (*p* = 0.01), and (iii) level of education (*p* = 0.04).

**Conclusion:**

In-degree centrality was the most important network characteristic. A culture of medication safety can be fostered by encouraging consultation about the medication of in-patients within the hospital network where reporting of medication errors is essential.

**Electronic supplementary material:**

The online version of this article (10.1186/s12913-018-3049-2) contains supplementary material, which is available to authorized users.

## Background

Patient safety is defined as the prevention of errors and adverse effects on patients related to their health care [1] and is an important aspect of healthcare policy worldwide [2–4]. Close attention to patient safety associates with lowered incidences of adverse events in hospitals [5], while a poor safety culture leads to increased error rates [6].

Previous studies demonstrated that poor communication about patient medication led to potentially harmful medication errors [7–9]. Miscommunication about medications (e.g., medication errors) within social networks of healthcare professionals [10, 11] including the effective reporting of errors has been an indicator that impinges on patient safety in healthcare settings [10] but also has provided enough information to develop an effective risk-management plan [12].

Social network analysis reveals details about complex communications and interactions between members in a network [13, 14] as applied to various healthcare settings (e.g., primary care, or hospital wards) [15, 16]. In pharmaceutical treatments, a unique pattern of social network in each healthcare setting can steer behaviours within organizations towards risk reduction in such environments. We therefore sought to examine characteristics of communication in the form of consultation about medication within social networks that are found in hospital inpatient medication systems, and their associations with medication error reporting.

## Method

### Design, and study setting

A mixed methodology was employed: (i) a prospective observational study to investigate incidences and the nature of reporting medication errors; (ii) a social network analysis of patterns of interactions within a hospital medication system.

This study was undertaken within an inpatient department of a 90-bed district hospital in Thailand, which included male and female general medical wards, an intensive-care unit (ICU), and a private medical ward. The average occupancy rate was ~ 60 inpatients during the study period.

In this hospital, the flow of the medication process began with (i) prescribing (physicians) by a hand-written entry on to the order sheet, (ii) pre-transcribing (nurses) by electronic scanning of the order sheet and transferring its information on to the pharmacy computer terminal, (iii) transcribing and appropriate labels pertaining to the medicine (pharmacists), (iv) pre-dispensing (pharmacist assistants) by filling the medicine pack as per label, and the pharmacist verifying accordance between the pack contents, the label, and the prescription, (v) dispatching the dispensed medications (pharmacists) to the appropriate ward (vi) pre-administration (nurses) to verify a match between the medication and the corresponding order sheet (vii) the medication administered (nurses) to the patient.

### Characteristic of participants

During the study period, 65 employees worked within the inpatient medication system: 7 physicians (1 medical specialist in internal medicine, and 6 junior physicians), 5 pharmacists, 44 nurses, 5 pharmacist assistants, and 4 unskilled ancillary workers.

### Data collection and analysis

To understand social networks in the medication system, this study required data on both reports about medication errors, and the consultation networks. Reports of these medication errors were collected at every stage of the medication process [17] and were graded as A to I as defined by the National Coordinating Council for Medication Error Reporting and Prevention (NCCMERP) [18]. Such error reports were collected for the 1-month period through the hospital spontaneous reporting system [19]. Staff were encouraged to report any event to the risk management committee. Reporters were anonymised using a code according to the protocol of data collection on consultation networks. On completing the data gathering, the reports were analysed using descriptive statistics.

Data on consultation networks in the medication system were collected from all 65 hospital participants by a structured face-to-face interview in a designated room to recall the previous months data collection period, in which data on medication errors were obtained. Two main questions were asked: (i) whether the participant consulted others about obstacles to medication use and medication-related problems. When the participant had consulted with others, they then provided the information about frequency of consultations based on recall over the last month; and (ii) who was consulted. (Additional file 1) Interview questionnaires were adapted from previous studies [15, 20] and validated by three experts in social network analyses and hospital medication systems. In this study, an ‘item objective congruence (IOC) index’ greater than 0.5 indicated an acceptable questionnaire content [21]. The information derived from the interview was then transformed to social network data (sociogram). Each pair of a relationship indicated a directed link from an informant to the consulting person in the network.

Social network data were analysed using UCInet version 6 [22] which provided both mathematical and visual analyses of network relationships as a sociogram representing complex intercommunication among hospital staff. Mathematic social network measures include the degree of centrality (in and out), and betweenness centrality. In-degree is a measure of the number of links from other staff directed to an informant, while out-degree indicates the number of links through which the informant sought consultations with other network members [15] or in lay terms, it indicates the extent to which he/she receives queries, or asked for opinion/advice. In this study, where the topic is about medication, those receiving more queries are believed to be expert about medication or are trustworthy within a network by other staff. Out-degree is the extent to which the informant seek advice from other staff. In this study, the questions were limited to the obstacles to medication use or medication-related problems; therefore, those who have out-degree possibly notice potential events, or have been involved in medication-related problems, or may hesitate to continue the process of medication use. Betweenness centrality was the degree of shortest path of the consultation seeking between all staff passing through the informant, calculated as the number of direct links from all staff of one group to all staff in another group to a specified individual. In lay terms, betweenness centrality is the extent that an individual influences the communication of other staff within the network. By passing the queries from one to another, those with high betweenness centrality seem to be a bridging person. An individual who provides a link between two different clusters demonstrates high betweenness centrality and is recognized as a ‘bridger’. Without a bridger, the network is disconnected. Therefore, finding bridgers is of importance because they represented those who often transfer information between groups and provide valuable opportunities for innovation [23].

Factors affecting the reporting of medication errors were analysed using linear regression [24] with the forward selection method using IBM SPSS Statistics version 23. Individual characteristics, in-degree, out-degree, and betweenness centrality were selected as the variables that influence reporting of medication errors. All statistical analyses were performed at a significance level of < 0.05.

## Results

### Characteristics of inpatient hospital staffs

Staff had an average age of 46.2 years (range 41–50) and were mostly female (*n* = 54; 83%). Healthcare professions included physicians (*n* = 7; 10.8%); nurses (*n* = 44; 67.7%), pharmacists (n = 5; 7.7%), pharmacist assistants (n = 5; 7.7%); and unskilled workers (n = 4; 6.2%). The majority of staff were educated to Bachelor level (*n* = 50; 77%).

### Characteristics of reporting medication errors

A total of 5296 prescriptions were dispensed and 132 medication errors were reported during the study period, an incidence of 2.5% of prescriptions. All medication error incidents were documented by pharmacists. Nearly all were judged as category B (*n* = 131; 99.2%), and one of category D. There were 54 pre-transcribing errors (40.9%), 28 prescribing errors (21.2%), and 20 pre-dispensing errors (15.2%). Medication errors tended to be higher in hospital wards (Table 1).

**Table 1:** Incidence rate, category of medication error, severity category and recognized reporter in each department

Department	Incidence rate, %	Category of Medication error			Severity category	Recognized reporter ID^c^
		Error category	N	(%)		
Pharmacy	0.84	Transcribing	18	(13.6)	B^a^	Pharmacists(2012, 2011, 2010)
		Pre-dispensing	20	(15.1)	B	
		Dispensing	7	(5.30)	B	
Specialty medical ward	1.51	Prescribing	3	(2.27)	B	Pharmacists(2012, 2011)
		Pre-transcribing	12	(9.1)	B	
		Pre-administration	1	(0.08)	B	
Ordinary female ward	1.66	Prescribing	9	(0.68)	B	Pharmacists(2012, 2010)
		Pre-transcribing	22	(16.7)	B	
		Pre-administration	1	(0.08)	B	
Ordinary male ward	1.84	Prescribing	14	(10.6)	B	Pharmacists(2012, 2011, 2010)
		Pre-transcribing	19	(14.4)	B	
		Pre-administration	2	(1.52)	B	
ICU	0.98	Prescribing error	2	(1.52)	B	Pharmacists(2012, 2011, 2009)
		Pre-transcribing	1	(0.08)	B	
		Administration	1	(0.08)	D^b^	
Totals	2.5	All errors	132	(100)		

^a^Category B: An error occurred but the error did not reach the patient; ^b^Category D: An error occurred that reached the patient and required monitoring to confirm that it caused no harm to the patient and/or required intervention to prevent harm; ^c^Recognized reporter is the person who reported the medical error, but not necessarily being the first to detect it

### Interactions among staff

**Fig. 1:**
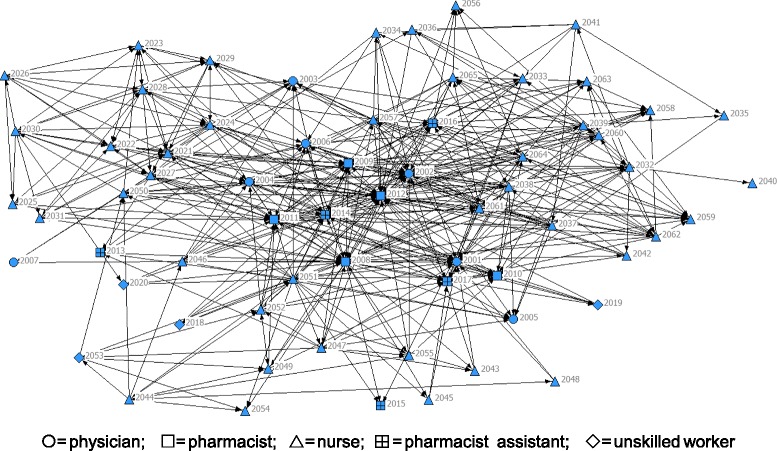
Sociogram of the inpatient medication network system (nodes represent persons; directed lines represent a consultation between two staff; ◯ = physician; ⃞ = pharmacist; ∆= nurse; ⊞ is = pharmacist assistant; ◇ = unskilled worker)

Consultation among the 65 staff showed that pharmacists played the greatest role in consultations indicated by a mean in-degrees centrality of 35.0 (SD = 14.9) while for physicians, it was 17.3 (SD = 12.5), 10.8 (SD = 6.4) for pharmacist assistants, 7.0 (SD =4.7) for nurses, and 4 (SD = 2) for unskilled workers (Table 2).Figure 1 illustrates networks of medication consultation among staff. Several arrows pointed towards the staff with IDs of 2009, 2010, 2011, and 2012 (squares), indicating high in-degree centrality and appeared to be recognized reporters (Table 1).

**Table 2:** Means of in-degree centrality and out-degree centrality for each type of profession from a workforce

Profession	Mean of in-degree centrality (S.D.)	Mean of out-degree centrality (S.D.)	Steps with highest in-degree^a^ (ID^b^; No. in-degree^c^)	**No. of reports**
Physicians	17.3 (12.5)	1.71 (2.4)	Prescribing (2002; 35)	0
Pharmacists	35.0 (14.9)	15.4 (11.1)	Transcribing (2012; 20) Pre-dispensing (2012; 10) Dispensing (2012, 18) Pre-administration (2012, 6)	132
Pharmacist assistants	10.8 (6.4)	10.8 (13.8)	–	0
Nurses	7.0 (4.7)	11.7 (9.3)	Pre-transcribing(2021, 4) Pre-administration (2022, 6; 2023, 6) Administration(2021,5; 2028, 5; 2029, 5)	0
Health workers	4.0 (2.0)	2.7 (0.6)	–	0
Whole network	10.4 (10.4)	10.4 (9.7)	–	132

^a^Steps in the medication system where each profession demonstrated the highest in-degree centrality; ^b^ID is anonymous code given to each person in the study cohort; ^c^Represent the highest in-degree in each step; in-degree centrality = a measure of the number of consultation the informant were asked for from other staff directed to an informant; out-degree centrality = the number of links that an informant sought consultation with other network members

The frequency at which consultations were sought about medications from other practitioners within the inpatient medication network were more evenly balanced between the health professions: mean out-degree centrality for physicians was1.71 (SD = 2.4); 15.4(SD = 11.1) for pharmacists, 11.7 (SD = 9.3) for nurses, 10.8 (SD = 13.8) for pharmacist assistances, and 2.7 (SD = 0.6) for unskilled workers. Table 2 shows the highest in-degree centrality for each profession in the medication system. By profession, (i) physicians had highest in-degree centrality in prescribing; (ii) pharmacists in transcribing, pre-dispensing, dispensing, and pre-administration, and (iii) nurses in pre-transcribing, pre-administration, and administration.

Two bridgers (participant IDs 2008 and 2012) were identified as important in the whole network by their high betweenness centrality (Table 3).

**Table 3:** Betweeness centrality for each participant. The cells in bold show two bridger participants

Paritcipant ID.	Betweenness centrality	Paritcipant ID.	Betweenness centrality	Paritcipant ID.	Betweenness centrality	Paritcipant ID.	Betweenness centrality
2001	0.0	2017	11.4	2033	25.1	2049	78.9
2002	150.9	2018	0	2034	6.7	2050	36.8
2003	0.0	2019	0.6	2035	0.7	2051	195.5
2004	44.0	2020	5.3	2036	16.1	2052	128.6
2005	0.0	2021	63.3	2037	35.4	2053	104.0
2006	28.9	2022	13.3	2038	32.4	2054	0
2007	0.0	2023	23.1	2039	123.5	2055	2.7
**2008**	**1291.0**	2024	167.7	2040	0	2056	0
2009	164.0	2025	13.1	2041	5.6	2057	163.3
2010	113.9	2026	1.2	2042	1.2	2058	1.2
2011	54.3	2027	88.4	2043	0.2	2059	1.2
**2012**	**1099.0**	2028	66.5	2044	57.6	2060	11.5
2013	0.0	2029	72.7	2045	0.2	2061	151.7
2014	186.9	2030	20.0	2046	12.7	2062	18.9
2015	0.0	2031	17.8	2047	224.2	2063	10.5
2016	174.8	2032	218.0	2048	1.3	2064	93.1
						2065	13.7

Betweeness centrality = the degree of shortest path of consultation seeking between all staff passing through the informant

### Associations between characteristics of consultation on medication and medication error reporting

Three factors: in-degree centrality (β = 0.7; *p* < 0.001), sex (β = − 0.3; *p* = 0.01), and education level (β = − 0.2; *p* = 0.04), were associated with reporting of medication errors (Table 4). Individual staff having high in-degrees centralities were linked to greater incidences of initiating reports of medication errorsespecially females. In addition, staff with a bachelor degree were more to report errors than staff graduating at higher degree levels.

**Table 4:** Linear regression analyses to predict the association between the characteristics of consultation and medication error reporting

Model	Unstandardized Coefficients		Standardized Coefficients	t^c^	*p*-value
	B^a^	SE	β^b^		
Constant	−5.4	1.9		−2.9	0.005
In-degree centrality	1.0	0.1	0.7	7.2	< 0.001
Sex	−9.6	3.7	−0.3	−2.6	0.012
Highest education level	−8.7	4.2	−0.2	−2.1	0.041

^a^B is the predicted regression coefficient; β^b^ is Standardized Coefficients; ^c^t is the t-statistic. The correlation coefficient (R^2^) for association between person reporting medication and characteristic of consultation is 0.46

## Discussion

This is the first study to examine the association between characteristics of social network and reporting among staff in the inpatient medication systems. Our findings revealed that pharmacists were central for medication consultation in the inpatient medication system of the whole network, which accords with their professional training and their recognition as experts in pharmacotherapy. This tallies with a previous study from a renal ward at an Australian metropolitan teaching hospital [15]. However, each profession played its own central role in different steps of the medication system. Regular updating of their knowledge and skills in medication use through continuing pharmacy education (CPE) programs is essential for safeguarding patients and making changes towards a safety culture in the process of medication use [25, 26].

Our findings about the role of bridgers in social networks has important implications to the development of organisations since a bridger represents a connector to other members of the network. They usually provide important information to others because this person is able to reach out and gain information from sub-networks better than other members in the network [23]. A bridger is perceived as an intermediary in the whole network for people seeking advice and to report any medication problems. In any healthcare setting, identification of bridgers, and support for their role will increase interactions among healthcare staff in medication consultation, thereby promoting a safety culture.

Three factors that significantly associated with reporting of medication errors were (a) in-degree centrality, (b) sex, and (c) level of education. In-degree was the most influential factor for reporting medication errors in the hospital, because staff with higher in-degree would report more medication errors than those with a lower in-degree. Noteworthy in our study, was that staff who first identified an error did not always directly enter the event into the system but rather consulted with a staff member having a high in-degree and appeared to be members of the risk management committee who were responsible for dealing with risks. This is probably because (i) staff who identify errors (‘whistle-blowers’) tend to worry about offending the co-worker and of reprisals [10] in spite of error reporting being specified in the guidelines and guaranteed anonymity; (ii) staff with high in-degree’s characteristics included both approachability by others and an official role according to the organizational structure. This reflects a hospital policy of only enforces the reporting of medication errors that have not been successfully implemented. Our findings strongly suggest that promoting consultation among staff including pharmacists provides greater error detection rates and minimises the risk of errors escaping detection. Thus, a positive working environment that deals with errors is thereby promoted and may lead to higher reporting of medication errors [27]. A qualitative study (e.g. focus group interview) should be performed to further confirm the reasons why errors are identified but fail to be reported.

Female staff showed higher reporting rates of medication errors than males. The explanation for this may be because most male staff in this study (physicians and pharmacy heads)had more managerial roles than front line staff who are more likely to be female (eg, nurses, pharmacists).

Staff at bachelor level appeared to report more medication errors compared to those with either higher or lower education levels: high educational achievement again equated with more managerial roles, while low attainment was associated with unskilled front line staff (eg, pre-packing medications or packing medications as labelled) who were not required to double-check labelling, etc., and thus unlikely to detect errors.

This study has much strength. All health professionals and workers contributed thus representing the whole network. Interviews using a validated questionnaire in a dedicated private room was employed to gain complex details of interaction between relevant staff. This contributes to a better quality of network data [28, 29]. Recall bias was minimized by asking staff about the consultations with colleagues on medications during just the previous month, whereas asking after 12 months of experiences may increase the probabilities of biases [15, 20]. This study also applied a sociocentric data collection methodology to assess the overall network performance rather than pinpointing individuals.

There were some study weaknesses. Although all staff were involved, the participant complement of the network was rather small which may contribute to less variability of reporting or reduce the reliability of the linear regression. In addition, this study could only detect errors found by recognized reporters, while staff are reluctant to report their own errors. [10, 30]. Furthermore, future studies should also audit medical records. This study could not analyse egocentric data which otherwise reveals the root cause of error because it breaks anonymity and violates study ethics [31].

## Conclusion

In-degree centrality was the most influential characteristic for reporting medication errors in the hospital inpatient medication system. Both the discovery and reporting of medication errors can be improved by creating an environment where consultation about medication regimes are promoted between members of a network and with pharmacists.

## Additional file

Additional file 1: Interview guide for consultation networks in the medication system. (DOCX 12 kb)

## Abbreviations

ICU: intensive care unit
NCCMERP: National Coordinating Council for Medication Error Reporting and Prevention
