# Supplementary material for: Analysis of medication consultation networks and reporting medication errors: a mixed methods study
Source: BMC Health Serv Res. 2018 Mar 27;18:221. doi: 10.1186/s12913-018-3049-2 (PMC5872530; doi:10.1186/s12913-018-3049-2)
Supplement: Supplementary file 1 — Interview guide for consultation networks in the medication system. (DOCX 12 kb) [file 12913_2018_3049_MOESM1_ESM.docx]

**Interview guideline** **for consultation networks in the medication system**

Analysis of medication consultation networks and reporting medication errors: A mixed methods study

Two main questions:

1. whether the participant consulted others about obstacles to medication use and medication-related problems. When the participant had consulted with others, they then provided the information about frequency of consultations based on recall over the last month; and
2. (ii) who was consulted.
